# Poly(ε-caprolactone) Scaffolds Fabricated by Melt Electrospinning for Bone Tissue Engineering

**DOI:** 10.3390/ma9040232

**Published:** 2016-03-25

**Authors:** Sascha Zaiss, Toby D. Brown, Johannes C. Reichert, Arne Berner

**Affiliations:** 1 Institute of Health & Biomedical Innovation, Queensland University of Technology, Brisbane, Queensland 4001, Australia; sascha.zaiss@t-online.de (S.Z.); toby-dbrown@gmail.com (T.D.B.); Johannes.Reichert@pgdiakonie.de (J.C.R.); 2 Department of Trauma Surgery, University Hospital Regensburg, Regensburg 93055, Germany; 3 Department of Orthopedics and Trauma Surgery, Evangelisches Waldkrankenhaus Spandau, Berlin 13589, Germany

**Keywords:** melt electrospinning, osteoblast, tissue engineering, bone, ovine

## Abstract

Melt electrospinning is a promising approach to manufacture biocompatible scaffolds for tissue engineering. In this study, melt electrospinning of poly(ε-caprolactone) onto structured, metallic collectors resulted in scaffolds with an average pore size of 250–300 μm and an average fibre diameter of 15 μm. Scaffolds were seeded with ovine osteoblasts *in vitro*. Cell proliferation and deposition of mineralised extracellular matrix was assessed using PicoGreen^®^ (Thermo Fisher Scientific, Scoresby, Australia) and WAKO^®^ HR II (WAKO, Osaka, Japan) calcium assays. Biocompatibility, cell infiltration and the growth pattern of osteoblasts on scaffolds was investigated using confocal microscopy and scanning electron microscopy. Osteoblasts proliferated on the scaffolds over an entire 40-day culture period, with excellent survival rates and deposited mineralized extracellular matrix. In general, the 3D environment of the structured melt electrospun scaffold was favourable for osteoblast cultures.

## 1. Introduction

Tissue engineering (TE) unites principles of engineering with biology to develop constructs that restore, maintain or improve tissue functions [1]. One key area of TE research is the production of porous materials (scaffolds) to provide three-dimensional (3D) support for cell migration, proliferation and differentiation [2]. TE scaffolds are made from biocompatible materials to promote cell adhesion, cell migration as well as cell invasiveness and provide sufficient mechanical strength and stiffness to allow a certain amount of movement in the damaged tissue [3]. Ideally, the scaffold fabrication process should allow systematic alteration of scaffold design to ensure a customisable and individualised scaffold architecture depending on the desired cell or tissue type [4]. Multiple scaffold fabrication processes exist, each with their own advantages and disadvantages in processing and biocompatibility [5]. Conventional scaffold fabrication methods including particulate leaching, gas foaming, solvent casting, phase separation and solution electrospinning are based on chemical processes and lack sufficient control over pore size, pore geometry and pore distribution to control cell–scaffold interactions [6]. Above mentioned fabrication methods use organic solvents to dissolve synthetic polymers resulting in concerns regarding cell toxicity and carcinogenic potential [7].

Recently, additive manufacturing (AM) approaches to fabricate scaffolds allow improved control over pore size and distribution [8]. AM is a collective term for a number of fabrication processes in TE that create 3D scaffolds in a layer-by-layer approach following a computer programmed design [9]. Widely used AM processes in TE include 3D inkjet printing, selected laser sintering, stereolithography and melt-extrusion based fused deposition modelling (FDM) [10]. FDM allows accurate control over scaffold architecture and properties, however, the relatively high viscosity of the polymer melt restricts the ability to be extruded through a small diameter nozzle, where the fibre diameter achievable is restricted to 100 μm [5]. Significantly lower filament resolutions as low as 0.5–10 μm can be reached in AM 3D colloidal inkjet printing [10,11]. However, the generation of clinically-relevant biomaterials from such colloidal inks is difficult from a regulatory perspective [11].

An alternative scaffold fabrication technique in TE that achieves small diameter filaments is electrospinning [12]. Electrospinning is based on electrohydrodynamic principles relying on an electrified viscous fluid jet being drawn through the air towards a collector at a different electric potential [13]. While solution electrospinning dominates the research area, there are an increasing number of papers on melt electrospinning, where a fibre is solidified by cooling rather than solvent evaporation [13]. Solution electrospun fibre diameters typically vary between 2 nm and several micrometres and provide high specific areas for cell attachment [12]. However, the chaotic nature of fibre deposition in solution electrospinning results in tightly packed non-woven meshes with pore sizes too small for cell penetration serving rather as substrates than as 3D scaffolds [14,15]. The use of volatile and often toxic solvents is another challenge of solution electrospinning scaffold production and should be removed before use [7]. To increase cell-invasiveness in electrospinning, several methods such as retrieving fibres collected onto water (hydrospinning) [16], salt leaching [17] and electrospinning onto ice crystals [18] have been developed. In addition, structured collectors have been used to produce open pore scaffolds. Whenever such approaches are employed, however, the mechanical strength is also greatly diminished. In order to achieve large pore sizes, bimodal scaffold production techniques combining solution electrospinning with larger scale AM fabrication methods to increase cell infiltration have recently been investigated [19]. Recently, a combination of solution electrospinning and melt electrospinning to produce a 3D cell-invasive scaffold has been described [20].

While melt electrospinning produces sub-micron diameter fibres, it can also result in much larger sized filaments than solution electrospinning, up to 250 μm [4,21]. A slightly larger, low micron diameter fibre should allow improved mechanical handling when the pore size is increased. Direct writing with melt electrospinning has recently shown that scaffolds with porosities as high as 98% can be readily handled [22]. Fibres can be stacked on top of each other (*i.e.*, electrostatic repulsion from previously deposited fibres is minimal) to produce true 3D scaffolds [23]. To ensure both cell infiltration and vascularisation in bone TE, melt electrospun scaffolds need to provide highly ordered and sufficiently large pores >100 μm [24]. Recent studies have demonstrated the ability to produce TE scaffolds with a pore size >100 μm to allow vascularisation through collector modifications [17,25].

This paper details melt electrospinning poly-(ε-caprolactone) (PCL) in a static way onto structured metallic collector substrates to produce open pore morphologies without using direct writing. Microscale and biocompatible 3D scaffolds with suitable pore sizes and fibre diameters for cell penetration with osteoblasts as bone forming cells were manufactured. Cell viability, cell morphology and growth pattern as well as proliferation and extracellular matrix (ECM) deposition of osteoblasts were investigated using scanning electron microscopy (SEM), confocal laser microscopy, proliferation and calcium assays *in vitro* to assess the biocompatibility of the melt electrospun PCL scaffolds and their suitability for bone TE applications.

## 2. Results

### 2.1. Scaffold Fabrication and Characterisation

**Figure 1:**
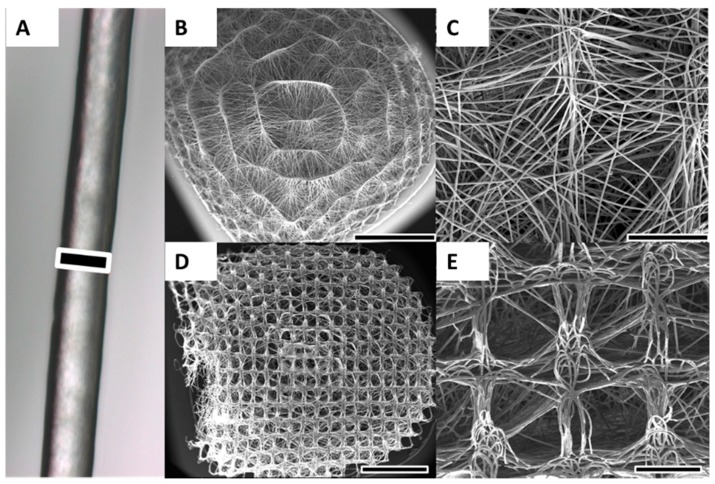
Melt electrospinning of PCL was used to produce 3D scaffolds. (**A**) Optical microscopy image of a representative melt electrospun PCL fibre; (**B**,**C**) Due to structured and curved metallic collector substrates, the scaffold possessed a convex-shaped architecture on the upper side of the scaffold. The average pore size on the convex side of the scaffold was 20–80 μm; (**D**,**E**) On the concave underside of the scaffold that was orientated towards metal collector substrate, the average pore size was 250–300 μm and matched the architecture of collector mesh. Scale bars: 15 μm (**A**); 2 mm (**B**,**D**); and 500 μm (**C**,**E**).

Melt electrospinning successfully produced homogenous batches of scaffolds with an average diameter of 6 mm and an average thickness of 3 mm (Figure 1B,D). The average fibre diameter was approximately 15 μm (Figure 1A). The fibres were smooth and without defects and the scaffold had significant pore interconnectivity. The average pore size varied between the concave scaffold underside contacting the metallic collector substrate during collection process and the convex-shaped top side. The average pore size on the concave side varied between 250 and 300 μm and matched the collector mesh architecture (Figure 1D,E). Average pore size on the convex-shaped top side was smaller and varied between 20 and 80 μm (Figure 1B,C). The performed NaOH treatment did not significantly affect pore size or fibre diameter, as shown in previous experiments from the Hutmacher group [26]. The scaffolds could be easily handled without breaking or folding, although pressure applied with a forceps was able to deform the scaffolds (Figure 1D, left side). In general, the scaffolds rebounded to their original shape, even with significant deformation.

### 2.2. Cell Proliferation and Calcium Deposition

**Figure 2:**
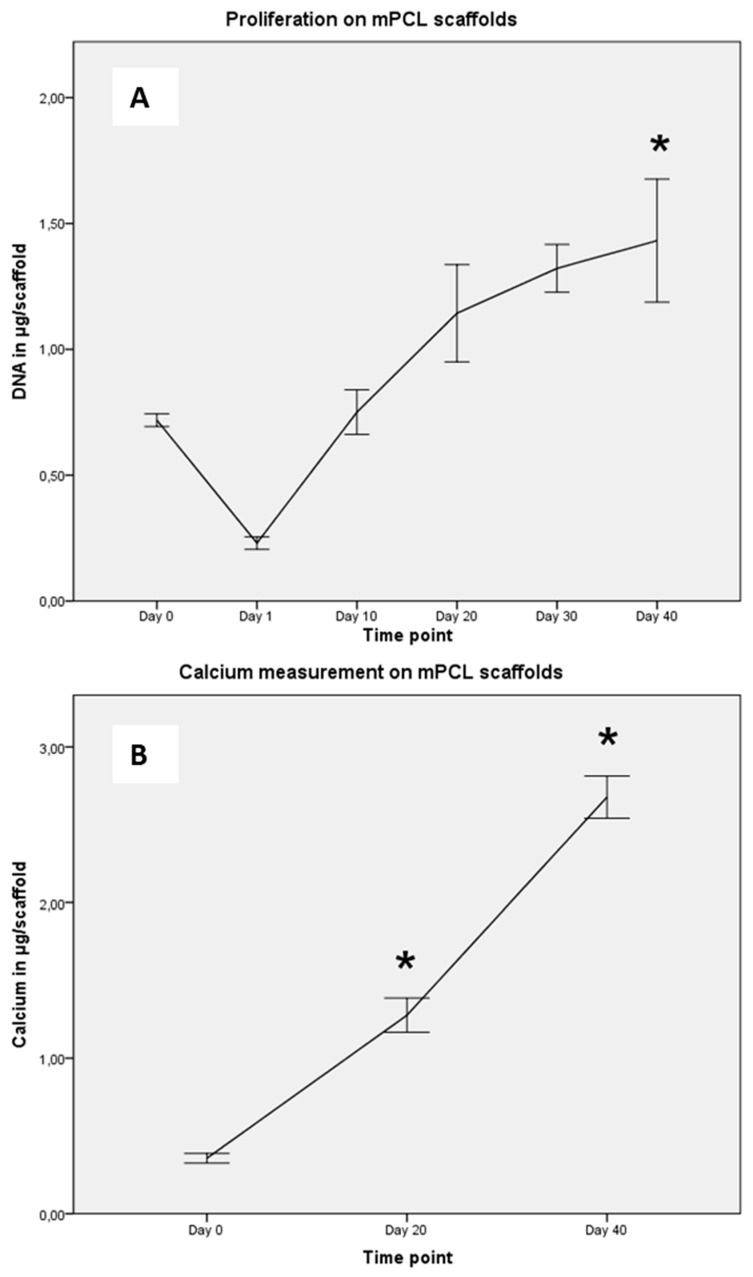
(**A**) Proliferation potential of osteoblasts (n = 6) was determined on Days 0 (seeding), 1, 10, 20, 30 and 40. Approximately one-third of seeded cells attached to the scaffold (Day 1). Osteoblasts proliferated over 40 days on scaffolds and the number of cells after 40 days was significantly higher than at Day 0 (*p* < 0.05) as measured in µg DNA per scaffold. A plateau-like phase was reached after 20 days; (**B**) Extracellular matrix deposition of osteoblasts was measured with significantly more calcium deposited at later time points than at seeding (*p* < 0.05). Intra- and extracellular calcium was measured in µg/scaffold and showed a linear fashion.

Osteoblasts were introduced onto PCL scaffolds using a static top seeding method onto the convex-shaped top side of the scaffold, with approximately a third of the seeded cells attaching to the scaffold after Day 1 (Figure 2A). These osteoblasts proliferated over the full 40-day culture period in osteogenic differentiation media. After 20 days, the cells reached a plateau-like phase showing a slower increase in DNA values per scaffold than before. After 40 days, the scaffold was populated by a significantly higher number of cells than at Day 0 (*p* < 0.05).

The concentrations of intra- and extracellular calcium deposition (measured in ng/scaffold) from osteoblasts on PCL scaffolds was determined using a WAKO^®^ HR II calcium assay and is shown in Figure 2B. Calcium desposition increased over time and at 20 and 40 days of culture in osteogenic differentiation media, the cells had produced significantly more mineralised ECM than at Day 0 (*p* < 0.05).

### 2.3. Cell Morphology, Viability and Growth Pattern

**Figure 3:**
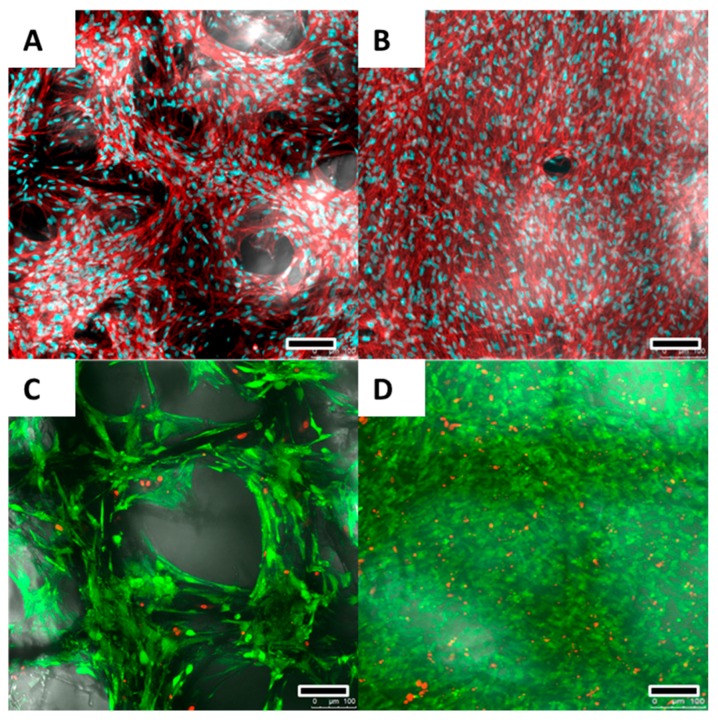
After 20 (**A**) and 40 (**B**) days, Actin/nuclei (Phalloidin/PicoGreen^®^) staining revealed an elongated and spindle-shaped morphology for the osteoblasts. The nuclei appear blue while actin cytoskeleton is visualised red; After 20 days (**A**,**C**), cell ingrowth into the scaffolds following the scaffold fibres could be observed with pores not yet completely bridged; After 20 (**C**) and 40 (**D**) days, live/dead cell staining revealed a cell viability of >90%. Alive cells are stained green while dead cells are being shown red. Scale bars: 100 μm (**A**–**D**).

**Figure 4:**
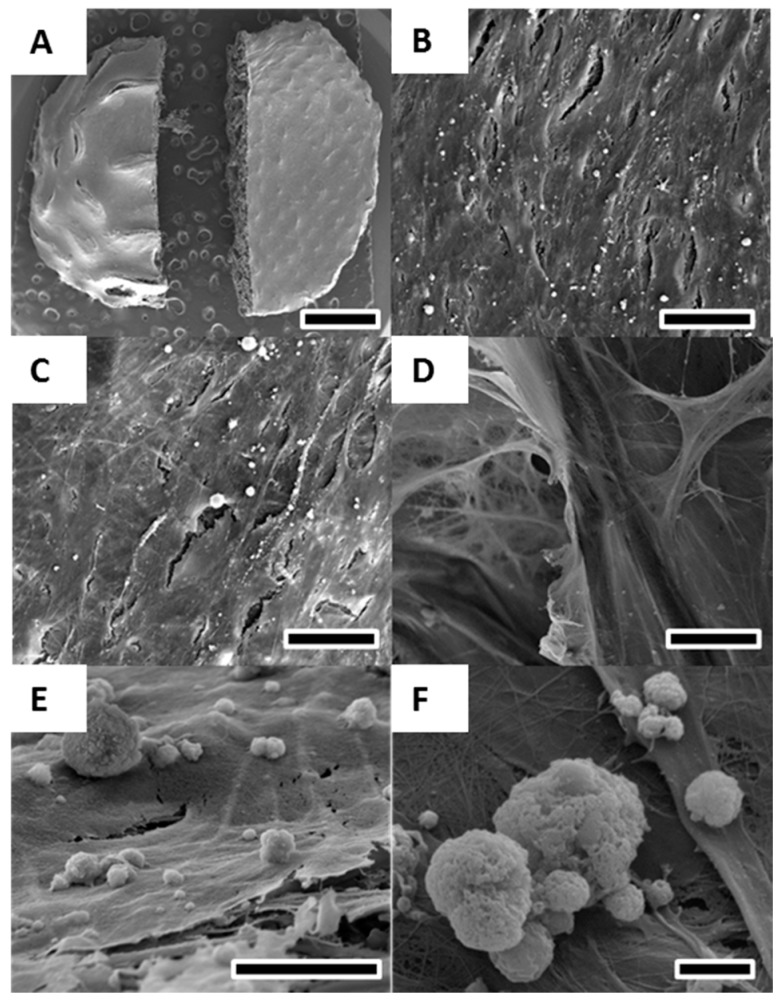
SEM of osteoblasts cultured for 40 days in osteogenic differentiation media on melt electrospun scaffolds. (**A**) Overview revealed a solid layer of osteoblasts covering the entire scaffold on both the convex (**B**) and concave (**C**) side; (**D**) Cell detection and calcium deposition on the inside of the scaffold demonstrating infiltration of cells into the porous scaffold structure; (**E**,**F**) High resolution image of calcium deposits formed by osteoblasts on the cell layer surface. Scale bars: 2 mm (**A**); 25 μm (**B**–**D**); 10 μm (**E**); and 2 μm (**F**).

The osteoblast viability, morphology and growth on PCL scaffolds was investigated after 20 and 40 days of culture by confocal laser microscopy using live/dead cell staining (Figure 3C,D) and actin/nuclei staining (Figure 3A,B) and SEM (Figure 4).

At both 20 and 40 days of culture, cell viability as assessed via FDA/PI dead-live cell staining showed >90% living cells (Figure 3C,D).

Actin/nuclei staining revealed an typical elongated, spindle-shaped, osteoblast like morphology (Figure 3A,B).

After 20 days, cell ingrowth into the scaffold following individual fibres could be observed while pores were not yet completely filled (Figure 3A,C). However, after 40 days, the scaffold surface was entirely covered with osteoblasts (Figure 3B,D).

SEM after 40 days of culture in osteogenic differentaition media revealed, similar to confocal laser microscopy, a spindle-shaped and elongated cell morphology of osteoblasts (Figure 4B,C). The scaffold was completely covered with a thick layer of osteoblasts (Figure 4A). Cell layer formation of osteoblasts did not differ between the large-pored concave scaffold side and the convex-shaped scaffold side with smaller pores (Figure 4B,C). Cell infiltration into and deposition of ECM on the inside of the scaffold could be observed (Figure 4D) and the evidence is supported by Actin/nuclei staining at Day 20 (Figure 3A). Osteoblasts deposited mineralised ECM on the scaffolds as visualised by Figure 4E,F. The sizes of the calcium deposits varied between 0.5 and 15 μm (Figure 4F).

## 3. Discussion

The suitability of scaffolds for TE applications depends on multiple parameters including fabrication method, fibre diameter, employed polymer, pore size, porosity, mechanical properties and pore geometry [27]. Scaffolds in this study were prepared by melt electrospinning a biodegradable polymer with a history of clinical use onto structured metallic collectors with the aim to provide optimal cell culture conditions for osteoblasts for bone TE applications. PCL is a linear polyester with a melting temperature of 60 °C, long degradation kinetics and can be readily blended with other polymers. As a result of this, and its adaptability to different processing technologies, PCL has been widely used in TE applications [27,28].

The average fibre diameter of fabricated scaffolds in this study was determined to be 15 μm and fibre structure was observed to be smooth and uniform with a homogenous fibre surface. Melt electrospun fibre diameters in other studies range from 270 nm to 350 μm, so there is the capacity to significantly modulate the fibre [21,23,29]. Due to extrudate die swell, fibre diameters in AM approaches such as FDM are a magnitude larger than melt electrospun fibres with a typical diameter of 100 μm [30]. Fibre diameters in solution electrospinning vary between 2 nm and several micrometres and are typically in the nanometre range [12,31]. The ideal fibre diameter for TE applications is controversially discussed. Recently, a number of groups have found superior cell proliferation on fibres in the low micrometre range compared with nanofibres [15,32,33]. Badami *et al.* [33] used electrospinning to produce scaffolds from different polymers with fibre diameters ranging from 0.14 to 2.1 μm. Following scaffold seeding with osteoprogenitor cells, an increased proliferative potential was observed on microfibres compared to nanofibres while cell adhesion was increased on nanofibres due to the large surface area [33]. The larger diameters of melt electrospun fibres offer the potential to create truly 3D structures with increased pore sizes for cell invasiveness, so far unachievable using nanoscale methods as solution electrospinning [34]. Random fibre deposition in solution electrospinning results in pore sizes too small for adequate cell penetration, therefore being perceived as 2D structures by cells [14]. AM approaches such as FDM with large fibre diameters of 100 μm are although initially perceived as 2D structures by cells, while a 3D effect is only observed after a certain time of cell culture by pore spanning [10]. Hence, melt electrospinning offers a promising approach to bridge the gap between nanoscale production methods with insufficient fibre deposition control such as solution electrospinning and resolution-limited AM approaches like FDM.

Melt electrospinning allows FDA approved polymers such as PCL to be processed in their pure form without the use of toxic solvents as in solution electrospinning or FDM [7,26]. Hence, potential approval times can be reduced and post-production costs to remove cytotoxic remnants are redundant. A disadvantage of PCL, however, is the poor wettability of the polymer due to its hydrophobicity resulting in generally poor cell adhesion. To ameliorate surface properties of our scaffolds, chemical surface modification with sodium hydroxide was applied prior to seeding the scaffolds [27]. Ideal attachment conditions depend on multiple factors including cell type and surface modification method to achieve the required hydrophile surface environment [26,35]. Chemical surface modification with sodium hydroxide is the most common and cheapest method available [26], reducing hydrophobicity through introduction of hydroxyl- and carboxyl-groups into the mPCL side branches, therefore increasing fibre surface and ameliorating cell attachment [36]. The cells used in our experiments showed a satisfying attachment of approximately 34% of initially seeded cells. Other groups using PCL scaffolds report significantly higher attachment rates than in our experiments [37]. Kim *et al.* reported attachment rates of 60%–80% for a bimodal collagen scaffold combining an AM with a nanofibre fabrication approach to increase cell adhesion [37]. Beside the known higher attachment rates of scaffolds produced with nanoscale fabrication methods, different surface modification methods for mPCL have emerged offering promising approaches to increase scaffold surface and cell adhesion. These include plasma treatment of mPCL scaffolds [38], blending biologically active materials such as bone morphogenic protein, hydroxyapatite, calcium phosphate or bioactive glass particles with PCL prior to electrospinning [39,40] and coating of electrospun PCL meshes with proteins such as laminin or collagen post electrospinning [41]. For future experiments with osteoblasts in bone TE, particularly coating or blending methods with bone morphogenic protein or hydroxyapatite would be interesting to ameliorate cell adhesion, proliferation and differentiation of osteoblasts on melt electrospun mPCL scaffolds. In our study, scaffolds were produced by melt electrospinning a pure polymer and cultured with typical bone forming cells, osteoblasts. Recently published articles follow a more complex approach to produce scaffolds for bone TE [42,43]. Paşcu *et al.* melt electrospun silk fibroin and nanohydroxyapatite to produce biodegradable and biocompatible scaffolds for bone TE [42]. Pasuri *et al.* used electrospun hydroxyapatite fibres embedded in Matrigel and cultured osteoclasts and macrophages, giving credit to the complex cell–cell interactions between osteoblasts, osteoclasts and macrophages necessary to promote bone formation [43].

Biocompatibility, cell proliferation, mineralised ECM deposition and cell viability as investigated with confocal laser microscopy, DNA measurements, calcium measurements and SEM showed high viability of >90% for osteoblasts on PCL scaffolds, proliferation and deposition of ECM over the entire cell culture period and an infiltrative growth pattern.

Based on results from confocal microscopy and SEM, osteoblasts were able to interact with the 3 mm thick scaffold as a true 3D structure demonstrated by cell infiltration into as well as growth on the outer layer of the scaffold. The influence of fibre diameter on cell adhesion and proliferation of osteoblasts has not yet been investigated in literature. However, Chen *et al.* described for fibroblasts, in combination with nanofibres, that as fibre diameter increased, cellular adhesion and proliferation decreased but remained constant when fibre diameter was altered within the micron range, as in melt electrospinning [44].

Fibre diameter and deposition in melt electrospinning depend on several parameters that can potentially be varied throughout the production process for future scaffold development [26]. 

Instrument-based parameters play a crucial role in melt electrospinning, since, due to the field’s infancy, most apparatus are custom-made [4]. Instrument-based parameters influence the fibre diameter [45] and include temperature, applied voltage, flow-rate of the syringe pump, distance between orifice and collector as well as speed and direction of the collector stage [22,45]. Dasdemir *et al.* demonstrated that increase in applied voltage and reduction of orifice-collector distance resulted in decrease of fibre diameter [46]. Another study by Kim *et al.* showed that a decrease in temperature (due to increased polymer viscosity) as well as an increase of the flow rate resulted in fibre diameter increase [47].

Material-based parameters are dependent on the utilized polymer and include electric conductivity [48], molecular weight [49], melting temperature [26] and tacticity [50]. Among the material-based parameters, molecular weight has the greatest influence and weights of 40,000 to 80,000 g/mol are most suitable [4]. Lower molecular weight results in decreased viscosity of the molten product and subsequently a decreased fibre diameter [26].

The porosity and pore sizes in electrospun scaffolds are mainly dependent on the fibre diameter [51]. Porosity of melt electrospun fibres is generally high due to the microscale fibre diameters and varies between 80% and 90% [27]. A recent study of Farrugia *et al.* [17] used melt electrospinning to produce PCL-scaffolds for fibroblast culture in skin TE applications. With an average 7.5 μm fibre diameter and an average 46 μm pore size, porosity was measured to be 87% in this study [17]. Pore size and geometry are an important area of investigation within scaffold-based bone TE applications. Scaffolds utilised in this study possessed a transition in such pore parameters from the lower to the upper sides. The pore geometry on the lower, concave, side reflected the square shape of the metallic collector substrate and the average pore size was 250–300 μm. On the upper, convex side, pore geometry was much smaller due to the more random fibre deposition, being between 20 and 80 μm due to fibre stacking resulting of charge collection. The smaller pore size on the convex scaffold side prevented cells from “falling” completely through the scaffold onto the bottom of the well to increase cell adhesion.

In a previous study, we investigated the influence of pore geometry on bone formation in a calvarial scull defect model *in vivo* [52]. In this study, scaffolds were produced from PCL and tricalcium phosphate with different pore geometries of 0°/90° and 0°/60°/120° using additive manufacturing. After insertion of scaffolds into scull defects in rats, computer tomography and histology indicated higher bone formation in scaffolds with square pore geometry without statistical significance. Another study by Bidan *et al.* cultured murine preosteoblastic cells on hydroxyapatite plates with different geometries *in vitro* showing increased tissue formation at cross-shaped pore geometries compared to square- and star-shaped geometries [53]. The differences in pore geometry of our scaffolds with square pore geometries on the concave and random pore geometry on the convex side did not affect the cell growth pattern of osteoblasts as indicated by confocal microscopy and SEM. This finding is backed by a recent study comparing proliferation of murine osteoblasts on melt electrospun PCL scaffolds with orderly structure and pore geometry to scaffolds with disorderly structure and pore geometry, where they did not observe any difference in proliferative potential [54].

A further advantage of a scaffold with different pore sizes is the possibility to co-culture different cell types such as osteoblasts combined with chondrocytes or endothelial cells for TE applications. Co-culture of osteoblasts and chondrocytes [55] as well as osteoblasts and endothelial cells [56] has recently been described in literature and offers interesting new approaches in TE. The growth distribution pattern and ECM deposition of osteoblasts on our scaffolds as investigated was homogenous and cell-invasive proving no negative aspect of the different pore sizes on each scaffold side. Multiple studies describe ideal pore size for osteoblast cell culture *in vitro* to vary between 100 and 400 μm [37,54]. Hence, our melt electrospun PCL scaffolds fulfil the desired requirements for osteoblast culture as demonstrated above.

## 4. Materials and Methods

### 4.1. Scaffold Fabrication and Preparation

**Figure 5:**
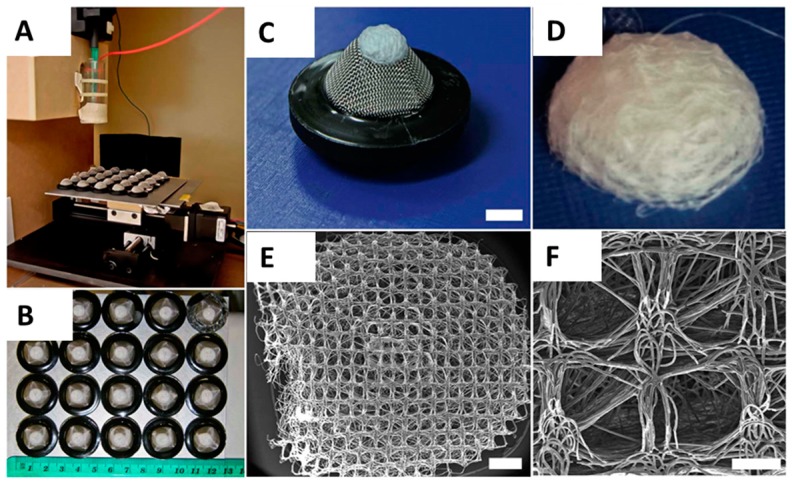
(**A**) Melt Electrospinning apparatus spinning molten PCL onto an array of 20 structured metallic substrates with raised architecture. X-y-stage moves substrates into path of melt electrospinning jet. Production parameters: Heating temperature 80 °C, flow rate 10 μL/h, applied voltage between needle and ground plate 20 kV, spinning duration per scaffold 20 min, needle-collector distance 50 mm; (**B**) Homogenous and replicable batches of porous PCL scaffolds; (**C**) Fibre deposition occurs preferentially onto raised surface of the patterned metallic collector substrate insulated with a rubber ring; (**D**) Fibre deposition creates a dome-shaped scaffold with a concave side towards the collector and a convex shape on the opposite side; (**E**) SEM image of concave scaffold side retaining the square-shaped porous pattern of the collector; (**F**) Average pore size of 250 μm on both concave and convex scaffold side. Scale bars: 5 mm (**C**); 1 mm (**E**); and 200 μm (**F**). Graphic reproduced from Brown TD *et al.* [4]. Materials Science and Engineering C 45 (2014) 698–708 with permission.

Melt electrospun scaffolds were fabricated with PCL (MW) 80,000 g/mol (Sigma Aldrich, Castle Hill, Australia), onto structured electroconductive collectors as described previously [4,17,57]. Briefly, PCL pellets were placed into a 3 mL Luer lock syringe (B-Braun, Bella Vista, Australia) and heated to 80 °C with the syringe held upright (Figure 5A). A blunt 23G needle attached to the syringe was used as a spinneret and placed into a water-circulating system heated to 80 °C. The PCL melt was electrospun using a spinneret-collector distance of 50 mm, a flow rate of 10 μL/h and a voltage of 20 kV applied to the spinneret. The melt electrospun PCL-fibres were collected onto an array of 20 dome-shaped wire mesh collectors upon a grounded moving plate. Each collector possessed a similar 250 μm × 250 μm square void template architecture with rubber rings around the base of each filter to provide a separating air gap to minimise disturbing influences on the jet’s path from the adjacent collector (Figure 5B,C). The steel mesh of each collector was the highest point on the collector and was positioned directly under the spinneret for the 20 min duration of fibre collection to produce a 3D scaffold (Figure 5C,D). Figure 5E,F reveals the heterogeneous structure on the concave of the scaffold, where the open square shaped 250 μm pores match the architecture of the collector mesh. After 20 min of fibre collection, an automated x-y-stage was used to move the molten PCL jet to the next collector.

The produced scaffolds were weighed, punched and microscopically analysed to ensure a consistent scaffold structure and mass. To ameliorate surface properties, PCL scaffolds were incubated with 5 M NaOH for 4 h at 37 °C, washed thoroughly with ddH_2_O and stored in 70% ethanol. Prior to experiments, scaffolds were incubated with fresh 70% ethanol for 2 h, transferred to 24-well tissue culture plates (Corning, New York, NY, USA) under a sterile work bench and UV sterilised for 30 min.

### 4.2. Cell Isolation

Ovine osteoblast explants were obtained from merino sheep (n = 6) (Ethic approval number 0900000099, animal ethics committee, Queensland University of Technology, Brisbane, Australia). Solid mandibular bone samples were acquired under sterile conditions, minced, washed with phosphate buffered saline (PBS, Invitrogen, Scoresby, Australia) and vortexed. After incubation with 10 mL 0.25% trypsin/ethyldiaminetetraacetic acid (EDTA, Invitrogen, Scoresby, Australia) for 3 min at 37 °C, 5% CO_2_ and trypsin inactivation with 10 mL low-glucose Dulbecco’s modified Eagle’s medium (DMEM, Invitrogen, Scoresby, Australia) containing 10% foetal bovine serum (FBS, Invitrogen, Scoresby, Australia), samples were washed with PBS once and transferred to 175 cm^2^ tissue culture flasks (Corning). Bone samples were topped-up with 15 mL of DMEM supplemented with 10% FBS and 1% penicillin/streptomycin (PS, Invitrogen, Scoresby, Australia). After 5–7 days, osteoblast outgrowth could be observed. Cells were expanded to second passage for subsequent experiments.

### 4.3. Cell Culture

A total of 50,000 osteoblasts were resuspended in 20 µL of DMEM/10%FBS/1%PS, seeded onto each PCL scaffold on the convex-shaped top side and incubated for 2 h at 37 °C, 5%CO_2_. After 2 h, 1 mL of basal media was added to each well of the 24-well plate. The following day, to prevent attachment of the scaffold–cell constructs to the bottom of the tissue culture plate, each scaffold was transferred to a 15 mL Falcon tube (Corning) using sterile tweezers and underwent osteogenic induction with 2 mL of DMEM/10%FBS/1%PS supplemented with 50 µg/mL l-ascorbic acid-2-phosphate, 10 mM β-glycerophosphate and 0.1 µM dexamethasone (all Sigma Aldrich). Scaffolds were further cultured at 37 °C, 5%CO_2_ in osteogenic media for up to 40 days.

### 4.4. Cell Proliferation

Triplicates of scaffolds seeded with osteoblasts were cultured with osteogenic media in 15 mL Falcon tubes for 1, 10, 20, 30 or 40 days at 37 °C, 5%CO_2_. At each termination point, scaffolds were washed twice with PBS, transferred to 1.7 mL Eppendorf^®^ tubes (Eppendorf, Macquarie Park, Australia), centrifuged at 500 rpm for 2 min at room temperature to remove excess liquid and stored at −80 °C until analysis. For analysis, scaffolds were digested with 50 µg/mL proteinase K (Sigma Aldrich) in 1× TE at 50 °C for 48 h. DNA content for 100 µL of each sample in triplicate were measured and quantified using a Quant-iT^™^ PicoGreen^®^ dsDNA assay kit (Thermo Fisher Scientific, Scoresby, Australia) according to the protocol supplied by the manufacturer (Invitrogen). An equal volume of the aqueous Quant-iT^™^ PicoGreen^®^ working solution was added to each triplicate. After three minute incubation on a rocking plate, fluorescence was measured at λ_excitation_ = 485 nm and λ_emission_ = 520 nm using a POLARStar OPTIMA plate reader (BMG Labtech, Ortenberg, Germany).

### 4.5. Calcium Measurement

To analyse the calcium content of the ECM produced by osteoblasts on the PCL scaffolds, a WAKO HRII Calcium assay (WAKO, Osaka, Japan) was performed according to the manufacturer’s protocol as described previously [58]. On Days 20 and 40, triplicate scaffolds were washed twice with ddH_2_O, centrifuged at 500 rpm for 2 min at room temperature (RT) and stored at −80 °C after removal of the supernatant. Samples were incubated with 500 µL of 10% v/v acetic acid for 3 h at RT and subsequently vortexed for 1 min at RT. To prevent evaporation, samples were covered with 200 µL of mineral oil (Sigma Aldrich), heated to 85 °C for 10 min and transferred to ice for 10 min. Following that step, the samples were centrifuged at 20,000 × G for 15 min. 300 µL of the supernatant were neutralized with 120 µL of 10% v/v ammonium hydroxide (Sigma Aldrich). 10 µL of the neutralized mixture were transferred in triplicate to transparent 96-well plates and incubated with 100 µL of monoethanylamine buffer, pH = 11 (WAKO, Osaka, Japan) for 3 min at 37 °C. Consequently, 100 µL of o-cresolphtalein-complex solution (WAKO, Osaka, Japan) was added to each well and incubated for 5 min at 37 °C. Plates were read at λ = 570 nm using a POLARStar OPTIMA plate reader (BMG Labtech, Ortenberg, Germany). To generate a standard curve, a serial dilution of Mulitchem Calibrator A (WAKO, Osaka, Japan) in 10% v/v acetic acid was used.

### 4.6. Confocal Laser Microscopy

Cell viability and morphology on PCL scaffolds was determined by staining with fluorescein diacetate (FDA) and propidium iodide (PI) or rhodamine-conjugated phalloidin (Phal) and PicoGreen^®^ (all Invitrogen) on Days 20 and 40. For FDA/PI staining, scaffolds were washed three times with phenol red-free media (Invitrogen) and incubated in dark for 15 min at 37 °C with an FDA/PI staining solution containing 2 µg/mL FDA, 10 µg/mL PI in phenol red-free media. As a cell-permeable esterase substrate, FDA is hydrolysed by viable cells giving green fluorescence. PI acts as a nucleic acid intercalator and penetrates the cell membrane of dead cells, but not living ones. Red colour was used to mark dead cells.

For Phal/PicoGreen^®^ staining, samples were washed three times with PBS, fixed with 4% paraformaldehyde (PFA, Sigma Aldrich) for 20 min at RT and washed two more times with PBS. Scaffolds were permeabilized with 0.2% Triton^®^ X-100 (Sigma Aldrich) in PBS for 5 min sharp at RT, washed twice with PBS and incubated with a staining solution consisting of 0.8 U/mL phalloidin, 1 µL PicoGreen^®^-Lösung/mL in 2% w/v BSA in PBS in dark for 50 min at RT. The cyclopeptide phalloidin binds to F-actin filaments of the cytoskeleton and therefore indicates cell morphology (red) while PicoGreen^®^ binds to dsDNA and therefore visualizes the nucleus (blue).

The stained scaffolds were washed three times with PBS to remove excess staining solution and visualized with a Leica SP5 confocal microscope (Leica Microsystems GmbH, Wetzlar, Germany).

### 4.7. Scanning Electron Microscopy

On Days 20 and 40, cell scaffold constructs were fixed with 3% v/v glutaraldehyde in 0.1 M sodium cocodylate buffer solution, pH = 7.3 for 2 h at 4 °C. The fixed specimens were dehydrated through a series of alcohols including two changes with 10 min in each 50%, 70%, 90% and 100% ethanol. Due to the PCL, samples could not be critical-point dried. Thus, remaining liquid was removed from the samples by incubating twice for 30 min using hexamethyldisalazane (Sigma Aldrich). Specimens were gold-coated in a SC 500 Bio-Rad sputter coater (Bio-Rad, Gladesville, Australia) and analyzed using a FEI Quanta 200 scanning electron microscope (FEI, Hillsboro, OR, USA).

### 4.8. Statistics

Statistical analysis was carried out using Student’s *t*-test and *p* < 0.05 was considered significant (SPSS).

## 5. Conclusions

The current study uses melt electrospinning of PCL onto structured metallic collector substrates to produce batch-to-batch similar scaffolds with an average fibre diameter of 15 μm and an average pore size of 250–300 μm on the concave side and 20–80 μm on the convex scaffold side. Biocompatibility, cell infiltration and growth of osteoblasts on PCL scaffolds was investigated using confocal laser microscopy with live/dead cell and actin/nuclei staining and SEM. Osteoblasts proliferated over the entire culture period, showed high survival rates and deposited mineralised ECM. Osteoblasts furthermore interacted with the mPCL scaffold as a true 3D environment as monitored by confocal microscopy and SEM. In the future, other bone forming cells such as osteoblasts derived from long bone or mesenchymal progenitor cells should be evaluated on the PCL scaffolds. In addition, understanding and correlating the *in vivo* performance of the scaffolds for bone TE applications should be undertaken.

## Abbreviations

The following abbreviations are used in this manuscript:

3D	three-dimensional
AM	additive manufacturing
ECM	extracellular matrix
FBS	foetal bovine serum
FDA	fluorescein diacetate
FDM	fused deposition modelling
PCL	medical grade poly-(ε-caprolactone)
PI	propidium iodide
PS	penicillin/streptomycin
SEM	scanning electron microscopy
TE	tissue engineering
